# Characterization of Sodium Alginate—Locust Bean Gum Films Reinforced with Daphnetin Emulsions for the Development of Active Packaging

**DOI:** 10.3390/polym14040731

**Published:** 2022-02-14

**Authors:** Hao Cheng, Jie Cao, Wenru Liu, Jun Mei, Jing Xie

**Affiliations:** 1 College of Food Science and Technology, Shanghai Ocean University, Shanghai 201306, China; m200300836@st.shou.edu.cn (H.C.); m190300743@st.shou.edu.cn (J.C.); m180300684@st.shou.edu.cn (W.L.); 2 National Experimental Teaching Demonstration Center for Food Science and Engineering, Shanghai Ocean University, Shanghai 201306, China; 3 Shanghai Engineering Research Center of Aquatic Product Processing and Preservation, Shanghai 201306, China; 4 Shanghai Professional Technology Service Platform on Cold Chain Equipment Performance and Energy Saving Evaluation, Shanghai 201306, China

**Keywords:** active film, physicochemical characteristics, daphnetin, scanning electron microscopy, antimicrobial activity

## Abstract

In this study, we characterized an active film made of sodium alginate (SA)—locust bean gum (LBG) containing daphnetin-based film. Physicochemical characteristics, as well as antioxidant and antibacterial properties, were investigated. The results showed that the addition of a low concentration of daphnetin increased the flexibility of SA–LBG cling film, leading to an improvement in elongation at break and tensile strength. As the daphnetin content increased, solubility, brightness and transparency of the cling film decreased, and the moisture permeability increased. The antioxidant capacity and antibacterial activity of films with daphnetin were improved compared to those of the basal film. In addition, the cling film formed by adsorption had higher bacterial (*Shewanella putrefaciens* and *Pseudomonas fluorescens*) inhibition and antioxidant activity rates than direct film formation. The results indicate that the combination of daphnetin in SA–LBG film provides an active film with antioxidant and antibacterial properties, with potential for the development of food-grade packaging material.

## 1. Introduction

Packaging plays an important role in preventing spoilage and extending the shelf life of fresh food [1]. In recent years, with growing demand for healthy and high-quality food, packaging materials have been given a series of new functions, and the concept of active packaging has emerged. Active packaging is an alternative type of packaging with multiple functions, such as preventing food contact with moisture and oxygen, producing or removing flavor, inhibiting microbial growth, and improving sensory properties of food [2,3]. In addition, increasing demand for green and safe food packaging has led to the growing importance of natural green packaging materials. In natural polymers, polysaccharides, proteins and lipids are the basic materials for the preparation of biodegradable active cling films due to their abundant sources and renewable nature [4,5,6].

Sodium alginate (SA) is commonly found in brown algae, such as kelp and *Sargassum*, which is non-toxic and inexpensive. Furthermore, it has the characteristics of degradability, safety, moisture retention, film formation and biocompatibility [7]. As a natural polymer material, SA can be used for food coating and making edible films, which is expected to reduce the environmental pressure caused by plastic products [8]. Research has shown that the film formed by SA coating is brittle after drying, requiring further enhancement of the mechanical properties and barrier capabilities of SA film [9]. As a natural anionic linear polysaccharide, SA has good water solubility because its molecular structure has more carboxyl and hydroxyl groups. It is good for mixing and reacting with other substances in water, which provides conditions for improved performance [10]. Blending of SA with other polysaccharides can effectively improve the performance of composite films, such as cellulose nano whisker, starch and chitosan, etc. [11,12,13]. Locust bean gum (LBG) is a natural, non-starchy and non-ionic polysaccharide composed of mannose and galactose residues, of which the ratio of galactose to mannose is 1:4 [14]. Since LBG is non-ionic, its viscosity and solubility are not influenced by the pH of the liquid medium. The galactose side chains of LBG have a strong synergistic effect with other polymers, such as better stabilization and emulsification when used with agar, carrageenan and other matrices. The film-forming solution has reduced gelling ability and increased viscosity when preparing films, and the composite film exhibits good processing and mechanical properties [15,16].

The application of natural preservatives as active coatings has drawn increasing attention [17]. Daphnetin is a coumarin derivative, which has been reported to attract antimicrobial and antioxidant properties [18,19]. Liu et al. [20] studied the microbiological, physicochemical and flavor changes of turbot (*Scophthalmus maximus*) coated with a composite active coating of locust bean gum (LBG) and sodium alginate (SA) supplemented with daphnetin emulsions (0.16, 0.32, 0.64 mg/mL) and found that it was a potential alternative way to improve the quality of turbot during refrigerated storage. However, daphnetin is insoluble in water, so it needs to be made into emulsion and then added to the preparation of cling film to be effective. In this study, we mainly evaluated the characteristics of SA–LBG containing daphnetin-based film with different concentrations of daphnetin and different film-forming methods, including mechanical properties, optical properties, barrier performance, water solubility, SEM observation, antioxidant activity and antibacterial properties. We aimed to provide a novel method of active packaging, thus extending to improve the efficiency of films and food packaging.

## 2. Materials and Methods

### 2.1. Materials

Lecithin, glycerin, sodium alginate (SA) and locust bean gum (LBG) were acquired from Shanghai Aladdin Biochemical Technology Co., Ltd. (Shanghai, China). Sodium chloride, 2,2-diphenyl-1-picrylhydrazyl (DPPH, radical) and vaseline were acquired from Sinopharm Group Chemical Reagent Co. (Shanghai, China). Activated carbon, iron powder and barium chloride were acquired from Shanghai Macklin Biochemical Co., Ltd. (Shanghai, China).

### 2.2. Preparation of Active Film

Daphnetin emulsions were made referring to the approach of Talón et al. [21]. Amounts of 0.024 g, 0.048 g and 0.096 g of daphnetin (0.5 × MIC, 1 × MIC and 2 × MIC) and 1.5 g of lecithin were added to deionized water. Then, the solution was homogenized by a handheld high-speed homogenizer at 35,000 rpm until completely mixed to obtain 100 mL of emulsion. Then, 0.8% locust bean gum, 1.2% sodium alginate and 0.6% glycerol were combined with the emulsion and stirred at 60 °C until complete dissolution to acquire film-forming solution. It was then sonicated using an ultrasonic-assisted homogenizer at 20 kHz and 700 W for 20 min to acquire the solution, which was degassed under vacuum.

The active films were made referring to the methods of Rubini et al. [22]. (i) Film without daphnetin was marked as basal group (CK). (ii) Film forming by adsorption: 300 mL of film-forming solution was injected into glass molds and poured into 100 mL of daphnetin emulsion before drying. After drying, films were obtained and labeled as A-0.5, A-1 and A-2, respectively. (iii) Direct-formation film was marked as control. A volume of 100 mL of daphnetin emulsion mixed with 200 mL of film-forming solution was added to a 25 cm × 25 cm glass film apparatus until completely dry to obtain cling films. The films were labeled as D-0.5, D-1 and D-2, respectively.

### 2.3. Thickness Detection

Test points were randomly selected to identify the thickness of cling films with a spiral micrometer (Shanghai Minet Industrial Co., Shanghai, China).

### 2.4. Color Difference

Test points were randomly selected to confirm the L*, a* and b* values of cling films with a CR-400 Konica Minolta Colorimeter (Konica Minolta Holdings, Inc., Minato, Japan). Each measurement was taken in triplicate. The calculation formula of ΔE was:

(1) $$\Delta E=\sqrt{{\left({\Delta L}^{*}\right)}^{2}+{\left({\Delta a}^{*}\right)}^{2}+{\left({\Delta b}^{*}\right)}^{2}}$$

### 2.5. Measurement of Light Transmittance

A 3 cm × 1 cm cling film was applied to the inner wall of the cuvette, and absorbance value at 600 nm was determined by a UV spectrophotometer (Unico Instruments Co., Ltd., Franksville, WI, USA) [23]. Each measurement was taken in triplicate. The formula for calculating light transmittance was:

(2) $$\mathrm{Light}\;\mathrm{transmittance}=A_{600}/X$$

### 2.6. Determination of Mechanical Properties

The cling film was cut into 10 cm × 1.5 cm strips, and then an automatic tensile tester was applied to measure the tensile strength (TS) and elongation at break (EAB) of the film [24]. Each measurement was taken in triplicate.

### 2.7. Determination of Oxygen Transmission Rate

The oxygen transmission rate was measured with reference to the approach of Liu et al. [25]. A weighed bottle containing 3 g of deoxidizer (iron powder: activated carbon: sodium chloride = 0.5:1:1.5) was covered with cling film and placed in a saturated solution of barium chloride (90% relative humidity) for 48 h at 25 °C. Each measurement was taken in triplicate. The original and final weight were recorded, and the calculation formula was:

(3) $$\mathrm{OP}=\frac{m_{f}-m_{i}}{t\times A}$$

where m_f_ is the final weight (g), m_i_ is the original weight (g), t is the equilibration time (h), and A is the cling-film area (m^2^).

### 2.8. Determination of Water Vapor Transmission Rate

The water vapor transmission rate was determined with reference to the approach of Liu et al. [26]. The films were sealed on a circular test cup (25 mm diameter) filled with deionized water. The test cup was placed in a desiccator on silica gel (0% RH) at a temperature of 20 °C. The weight was measured at 24 h intervals for 7 days. Each measurement was taken in triplicate. The calculation formula was:

(4) $$\mathrm{WVP}=\frac{\mathrm{WVTR}\times L}{\Delta P\times A}$$

where WVTR represents the slope of the curve from weight loss versus time, ΔP is the difference in water vapor pressure across the cling film (kPa), and A is the film area (m^2^).

### 2.9. Determination of Water Solubility

The determination of water solubility referred to the approach of Wang et al. [27]. The films were dried to stable weight (W_i_) and dipped in 50 mL of deionized water for 15 h, and the insoluble material was dried. The dried residue was weighed (W_r_). Each measurement was taken in triplicate. The formula for calculating the water solubility of the membrane was:

(5) $$W_{s}\left(\%\right)=\frac{W_{i}-W_{r}}{W_{i}}\times 100\%$$

### 2.10. Determination of Antioxidant Activity

The DPPH (radical) method was used to determine antioxidant activity [23]. In Brief, 25 mg of sample was added to 3 mL distilled water. A volume of 2.8 mL of each sample’s extracted solution was mixed with 0.2 mL DPPH methanol solution (1 mM). The mixture was kept at 25 °C in darkness for 30 min. The absorbance of solution was read at 517 nm. Each measurement was taken in triplicate. DPPH antioxidant activity was calculated as follows:

(6) $$\mathrm{Antioxidant}\;\mathrm{activity}\;\left(\%\right)=\frac{A_{\mathrm{DPPH}}-A_{S}}{A_{\mathrm{DPPH}}}\times 100\%$$

where A_DPPH_ is the absorbance of DPPH methanol solution, and A_S_ is the absorbance of sample solution.

### 2.11. Determination of Bacterial Inhibition

Inhibition activity of cling film against *S. putrefaciens* and *P. fluorescens* was measured by the methods of Aguilar-Sanchez et al. [28]. A weight of 0.5 g of cling film was added to 50 mL of the bacterial solution and incubated at 30 °C with shaking for 4 h. Then, 100 μL of the solution was removed and applied on nutrient agar, then incubated at 30 °C for 24 h. A growth control was prepared in parallel to ensure that viable organisms were present. The number of each group growths was determined by counting and compared with the growth control. The results were expressed as percentage. Each measurement was taken in triplicate.

### 2.12. Scanning Electron Microscopy (SEM)

Surface morphologies were observed by a SU5000 scanning electron microscope (Hitachi, Japan). Prior to observation, the surface specimens were sputtered with gold.

### 2.13. Statistical Analysis

Data were processed by SPSS 24.0 statistical software and one-way ANOVA, and multiple comparisons were performed by Duncan’s test. Bar graphs were plotted with Origin 2021, and the results were expressed as mean ± SD.

## 3. Results and Discussion

### 3.1. Mechanical Properties of Cling Film

The effects of different concentrations of daphnetin and different film-formation methods on the thickness and mechanical properties of the cling film are shown in Table 1. There was no significant difference in the thickness of the composite cling films as the content of daphnetin increased. The adsorbed film-forming samples had the same thickness as the CK samples, and the control samples were thicker. When the cling film is used in food packaging, TS and EAB are usually applied to evaluate the mechanical properties of the films, which are important properties related to the strength and flexibility. As shown in Table 1, TS and EAB values of the CK film were 0.76 KN/m and 8.9%, respectively. In the directed film-forming treatment group, the D-1 samples had the highest TS and EAB values. In this study, it was found that low concentrations of daphnetin improved the flexibility of cling film, resulting in increased TS and EAB values, whereas the addition of higher levels had the opposite effect. The mechanical properties of cling films in groups A-0.5, A-1 and A-2 showed a declining trend with the increase in daphnetin content. Increased EAB suggested increased plasticization effects of the incorporated active ingredients, which improved molecular mobility and flexibility [29]. The results reveal that the addition of daphnetin significantly affected the mechanical properties of cling film, probably due to the effect of daphnetin on the structural organization and interactions constituting the gel network. When cross-linked too extensively, cling films can become fragile and easily broken [30]. Moreover, the structural changes caused by daphnetin are mainly due to non-covalent forces, such as hydrogen bonds between hydroxyl groups in daphnetin and acceptors in myofibril, as well as hydrophobic interactions between aromatic rings of daphnetin and aromatic amino acid residues [31]. Rubini et al. [22] also the effect of compared direct syntheses and adsorption methods on properties of films. The study found that direct synthesis in water/ethanol (50/50) provoked precipitation of quercetin in solution, and water/ethanol was used as a solvent following an adsorption procedure. The authors concluded that different film formation methods had a significant impact on the performance of films.

### 3.2. Optical Properties of Cling Film

**Figure 1:**
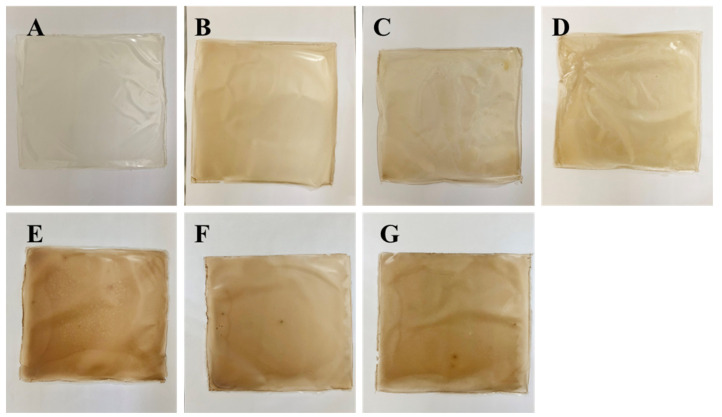
Differences in surface of films: CK (**A**), A-0.5 (**B**), A-1 (**C**), A-2 (**D**), D-0.5 (**E**), D-1 (**F**) and D-2 (**G**) films. CK: films without daphnetin; A: films formed by adsorption; D: direct-formation films; 0.5, 1, 2: 0.5 × MIC, 1 × MIC and 2 × MIC.

The color of cling film is an important index that affects consumer acceptance. Optical properties are shown in Table 2, and pictures of the films are shown in Figure 1. Visually, the color of the cling film was pale yellow and transparent because the film was enriched in sodium alginate, and the color deepened as the concentration of daphnetin increased (Figure 1B–G). The deepening of the film-solution color was caused by the direct addition of daphnetin to the SA–LBG solution. As the daphnetin concentration increased, the brightness (L*) of the cling film decreased, which meant that less light was reflected from the surface of the cling film. As the daphnetin content increased, the clarity of the cling film decreased, which meant less light transmission through the cling film, indicating a promising anti-ultraviolet property of the films. This delayed lipid oxidation and maintained the organoleptic properties of packaged foods, thus extending their shelf life. The higher chromatic aberration (ΔE) value also reflected the effects of polyphenols on the appearance of the cling film. The addition of daphnetin changed the optical properties of the film, which also improved the oxidation resistance of the film, and to some extent, it improved of the film’s properties. The appearance of the cling films seemed to be pale yellow compared to basal films. This was due to the addition of daphnetin; even though the color was changed, it improved function in food packaging. Obviously, it would take some time for consumers to accept this change.

### 3.3. Barrier Performance and Water Solubility

One of the major functions of cling film is to impede the transfer of water between food and the environment [32]. In general, water vapor permeability depends on the diffusion and dissolution of molecules in the cling-film base [33]. Compared to other films, D-2 and A-2 films exhibited higher water vapor permeability (Table 3). Daphnetin and biopolymer interactions formed a dense system of composite films, leading to greater water resistance. The addition of daphnetin improved oxygen barrier properties of the cling film, which was consistent with the development of a tighter, more enclosed structure. Increased hydrophilicity of the matrices reduced the oxygen permeability of the films, as oxygen is a non-polar molecule [34]. Moreover, Leelaphiwat [35] also showed higher plasticization with improved compatibility and dense matrix structure, which consequently reduced permeability of the films. However, high concentrations of daphnetin might agglomerate and create pores in the polymer matrix, leading to increased water vapor permeability [36]. In addition, it was found that the water solubility of direct-formed and adsorption-formed films was significantly lower than that of CK films. The addition of daphnetin led to the formation of hydrogen bonds between molecules, reducing free hydroxyl groups in the cling film and limiting the binding of hydroxyl groups to water molecules [37]. This effectively avoided the dissolution of hydrophilic molecules in water, which reduced the water solubility of the cling film and enhanced the intermolecular forces within the film. Wongphan [38] also indicated that hydrogen bonding between modified starch polysaccharides and papain eliminated free hydroxyl groups and reduced water dissolution. In addition, the hydrophilic group in the lecithin molecule improved the hydrophilicity of the cling film, and the addition of daphnetin might increase the level of bonding between molecules, reducing the solubility.

### 3.4. SEM Analysis

**Figure 2:**
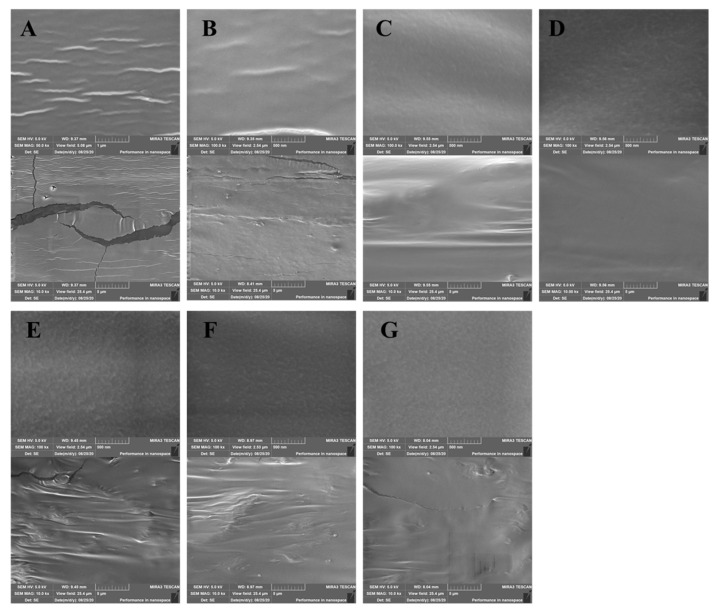
Differences in SEM surface micrographs of films: CK (**A**), A-0.5 (**B**), A-1 (**C**), A-2 (**D**), D-0.5 (**E**), D-1 (**F**) and D-2 (**G**) films. CK: films without daphnetin; A: films formed by adsorption; D: direct-formation films; 0.5, 1, 2: 0.5 × MIC, 1 × MIC and 2 × MIC.

The morphologies of the SA–LBG-based films observed with SEM is shown in Figure 2. The surface of the CK film (Figure 2A) appeared to be slightly rough and cracked, which might be attributed to the poor mechanical properties of the SA films [9]. In contrast, the surface of the film with daphnetin was smoother and more uniform (Figure 2B–G), indicating that daphnetin, sodium alginate and locust bean gum were uniformly mixed. The daphnetin did not sharply change the morphology of the SA–LBG films, indicating a good compatibility. Smooth microstructures indicated miscibility between the incorporated active compounds and the polymer matrices [39]. Compared to the cling film formed by adsorption (Figure 2B–D), a rougher surface was observed for the direct-formation film (Figure 2E–G), indicating better compatibility of the cling film formed by adsorption. Other authors have observed that polymer–phenolic (daphnetin) interactions give cling film denser and tighter properties [40].

### 3.5. Antioxidant Activity

**Figure 3:**
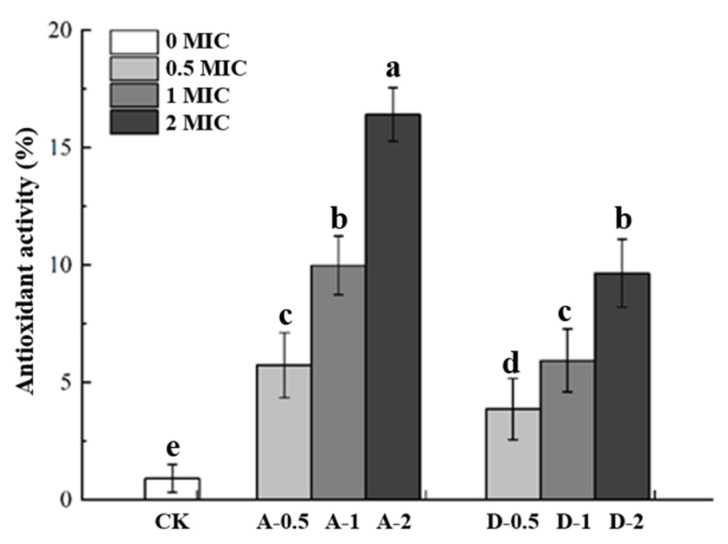
Differences in antioxidant activities of composite films. CK: films without daphnetin; A: films formed by adsorption; D: direct-formation films; 0.5, 1, 2: 0.5 × MIC, 1 × MIC and 2 × MIC. Different superscript letters (a, b, c, d, e) in the same column indicate significant differences (*p* < 0.05).

DPPH radical scavenging capacity is a measure of antioxidant activity. The higher the DPPH radical scavenging rate, the higher the antioxidant capacity of the cling film. Figure 3 shows the DPPH results for different cling films. Compared to the cling film enriched with daphnetin, the CK cling film had lower antioxidant activity. The antioxidant activity was significantly increased after adding daphnetin, and the degree of antioxidant capacity was proportional to the amount of daphnetin added (0.5%, 1% and 2%). In addition, the cling film formed by adsorption has better antioxidant capacities than CK films. This might be attributed to the release rate of different film-forming methods. The antioxidant activity of these cling films was mainly attributed to the antioxidant properties of daphnetin [19].

### 3.6. Antibacterial Properties

**Figure 4:**
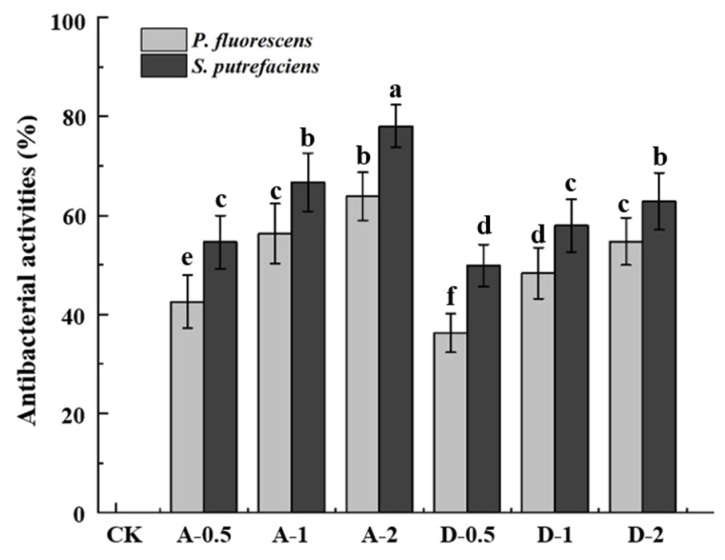
Differences in antibacterial activities of composite films. CK: films without daphnetin; A: films formed by adsorption; D: direct-formation films; 0.5, 1, 2: 0.5 × MIC, 1 × MIC and 2 × MIC. Different superscript letters (a, b, c, d, e, f) in the same column indicate significant differences (*p* < 0.05).

Antimicrobial activity of the active films depend on the release rates of the active ingredients [41]. In this study, the agar diffusion method was used to determine the antibacterial activity of daphnetin cling film against *S. putrefaciens* and *P. fluorescens*. Liu et al. [20] found that the minimum inhibitory concentration (MIC) of daphnetin was 0.08 mg·mL^−1^ for *Shewanella putrefaciens* and 0.16 mg·mL^−1^ for *Pseudomonas fluorescens*. As shown in Figure 4, the cling film of the CK group did not contain daphnetin and therefore showed no antibacterial activity. Since daphnetin has a strong antibacterial effect, the cling film containing daphnetin showed a good antibacterial effect. The results showed that for different concentrations of daphnetin cling film, the higher the daphnetin content, the better the antibacterial effect. For different strains, the cling film was more effective in inhibiting *S. putrefaciens* at the same concentration due to the higher antibacterial activities of daphnetin against *S. putrefaciens* [20]. Cling film formed by adsorption has a higher bacterial inhibition rate than direct-formation films. This might be due to the fact that the release of daphnetin into the bacterial solution was slower during direct film formation, delaying the effect.

## 4. Conclusions

In this experiment, the effects of different contents of daphnetin and different film-formation methods on the characteristics of active cling film were researched. The experimental results revealed that the addition of an appropriate content of daphnetin improved the optical properties, mechanical properties and barrier characteristics of the cling film. The addition of excessive daphnetin increased the pore space inside the cling film, which had a negative impact on physical indicators, such as tensile strength (TS), elongation at break (EAB), water evaporation transmission rate and oxygen transmission rate. With increased daphnetin content, solubility, brightness and transparency of the cling film decreased, and moisture permeability increased. The higher the content of daphnetin, the stronger the antioxidant activity and antibacterial activity of the cling film. The antioxidant and antibacterial effect of the sample made by adsorption was better than that of the sample made by direct film formation. The combination of daphnetin in SA–LBG films provides an active film with antioxidant and antimicrobial properties and has the potential to contribute to food packaging. Future research will focus on the development of different film-formation methods and decreasing the sensory impact of additions on the films.

## Figures and Tables

**Table 1:** Differences in mechanical properties of composite films.

Samples	Thickness (mm)	TS (KN/m)	EAB (%)
CK	0.101 ± 0.002 ^a^	0.76 ± 0.02 ^d^	8.9 ± 0.49 ^b^
A-0.5	0.103 ± 0.003 ^a^	2.07 ± 0.09 ^a^	11.3 ± 0.53 ^b^
A-1	0.101 ± 0.001 ^a^	1.81 ± 0.03 ^b^	10.4 ± 0.64 ^b^
A-2	0.098 ± 0.001 ^a^	1.75 ± 0.05 ^b^	9.9 ± 0.57 ^b^
D-0.5	0.113 ± 0.003 ^a^	1.31 ± 0.05 ^c^	18.7 ± 0.63 ^a^
D-1	0.110 ± 0.003 ^a^	1.53 ± 0.02 ^c^	19.2 ± 0.56 ^a^
D-2	0.112 ± 0.003 ^a^	1.40 ± 0.07 ^c^	17.2 ± 0.66 ^a^

CK: films without daphnetin; A: films formed by adsorption; D: direct-formation films; 0.5, 1, 2: 0.5 × MIC, 1 × MIC and 2 × MIC. Different superscript letters (a, b c, d) in the same column indicate significant differences (*p* < 0.05).

**Table 2:** Differences in optical properties of composite films.

Samples	Brightness (L*)	Degree of Red-Green (a*)	Degree of Yellow-Blue (b*)	Total Color Difference (ΔE)	Transparency (mm^−1^)
CK	−1.93 ± 0.14 ^c^	−0.85 ± 0.03 ^c^	+7.23 ± 0.30 ^c^	28.36 ± 2.64 ^f^	1.03 ± 0.03 ^e^
A-0.5	−11.34 ± 0.37 ^b^	+1.57 ± 0.11 ^b^	+15.69 ± 0.11 ^b^	188.62 ± 6.78 ^e^	2.78 ± 0.14 ^c^
A-1	−13.70 ± 0.39 ^b^	+1.53 ± 0.12 ^b^	+16.15 ± 0.45 ^b^	225.43 ± 11.92 ^d^	2.02 ± 0.04 ^d^
A-2	−15.65 ± 0.42 ^b^	+1.98 ± 0.08 ^b^	+24.64 ± 0.76 ^a^	427.99 ± 17.43 ^c^	1.93 ± 0.11 ^d^
D-0.5	−23.92 ± 1.25 ^a^	+5.76 ± 0.15 ^a^	+25.13 ± 0.68 ^a^	618.43 ± 21.97 ^a^	5.04 ± 0.07 ^a^
D-1	−23.40 ± 1.24 ^a^	+6.15 ± 0.21 ^a^	+21.59 ± 0.74 ^a^	525.76 ± 22.24 ^b^	5.09 ± 0.11 ^a^
D-2	−24.48 ± 1.27 ^a^	+6.13 ± 0.19 ^a^	+23.63 ± 0.83 ^a^	597.61 ± 22.51 ^a^	4.17 ± 0.12 ^b^

CK: films without daphnetin; A: films formed by adsorption; D: direct-formation films; 0.5, 1, 2: 0.5 × MIC, 1 × MIC and 2 × MIC. Different superscript letters (a, b, c, d, e, f) in the same column indicate significant differences (p < 0.05).

**Table 3:** Differences in barrier properties, water solubility and contact angles of composite films.

Samples	Water Vapor Permeability (g·mm/m^2^·kPa·h)	Oxygen Permeability (g/m^2^·h)	Water Solubility (%)
CK	11.20 ± 0.43 ^e^	8.11 ± 0.40 ^c^	0.349 ± 0.02 ^a^
A-0.5	13.83 ± 0.47 ^c^	8.41 ± 0.33 ^b^	0.287 ± 0.00 ^b^
A-1	14.24 ± 0.58 ^bc^	8.22 ± 0.43 ^b^	0.274 ± 0.02 ^b^
A-2	14.72 ± 0.52 ^ab^	7.93 ± 0.19 ^d^	0.285 ± 0.04 ^b^
D-0.5	11.46 ± 0.51 ^e^	8.68 ± 0.35 ^a^	0.284 ± 0.01 ^b^
D-1	12.83 ± 0.41 ^d^	8.65 ± 0.12 ^a^	0.274 ± 0.02 ^b^
D-2	15.25 ± 0.49 ^a^	8.30 ± 0.17 ^b^	0.213 ± 0.02 ^c^

CK: films without daphnetin; A: films formed by adsorption; D: direct-formation films; 0.5, 1, 2: 0.5 × MIC, 1 × MIC and 2 × MIC. Different superscript letters (a, b, c, d, e) in the same column indicate significant differences (*p* < 0.05).

## Data Availability

Not applicable.
